# A salutogenic approach to aspects facilitating female immigrants' establishment in working life: an integrative literature review

**DOI:** 10.1080/17482631.2025.2584750

**Published:** 2025-11-14

**Authors:** Cecilia Garell, Marie Lydell, Marie-Helene Zimmerman Nilsson, Krister Hertting

**Affiliations:** a School of Health and Welfare, Halmstad University, Halmstad, Sweden; b Division of Educational Science and Languages, Department of Social and Behavioural Studies, University West, Trollhättan, Sweden

**Keywords:** Facilitators, holistic health, salutogenesis, immigrant women, integrative literature review, working life

## Abstract

**Background:**

Studies show that female immigrants tend to struggle to establish themselves in the working life in a new society. Since previous research has primarily focused on barriers to integration, there is a need for more studies that encompass aspects that make integration toward employment possible for women.

**Purpose:**

This integrative literature review aimed to explore peer-reviewed research on facilitating aspects for immigrant women in their process of integrating into working life.

**Methods:**

An integrative literature review yielded 15 peer-reviewed articles published between 2010 and 2024.

**Results:**

The review revealed two categories of facilitators; the power of agency and social networks as prominent patterns in the reviewed articles. The agency appeared as an active endeavour to learn culture and language, supported by social networks from family, friends, and workplaces. However, there seems to be an imbalance between the discourse of integration as a two-way process and the idea of assimilating to dominant ideas of workplaces and society.

**Discussion:**

The authors discuss the implications of these findings concerning the different meanings of integration, inclusion, assimilation, and health.

## 1 Introduction

People migrate for different reasons, including economic, political, and/or social aspects (IOM, 2022). Coming to a new society is associated with complex processes of maintaining continuity and incorporating change into an immigrant’s life (Hertting & Karlefors, 2021) and is described as the process of becoming an accepted part of society (Penninx & Garcés-Mascareñas, 2016). Penninx and Garcés-Mascareñas (2016) point out that integration processes are multilayered with an inherent imbalance in power relations, driven by actors in the host society and involving legal/political, socio–economic, and cultural/religious processes. Immigrants are often expected to assimilate and adopt their behaviors, values, and beliefs (Malik & Manroop, 2017). At a political level, cultural diversity is respected, and integration is recognized as a two–way process between the immigrant and society. However, host society residents do not always accept responsibility in such a two–way integration process (Malik & Manroop, 2017).

This article focuses on successful integration as accomplishments in public spheres (such as working life), which serve as integration markers and means (c.f. Ager & Strang, 2008). According to Ager and Strang (2008), employment is a common marker for integration and an important pathway into a new society. It contributes to economic independence, learning language and culture, and health–promoting factors such as restoring self–esteem and increasing social connections (Ager & Strang, 2008; Moffitt et al., 2020). However, a challenge many immigrants face is acquiring employment matching their education and experiences from their country of origin. According to Ager and Strang (2008), this process can negatively affect health and feelings of belonging in the new society.

Thus, entering the labor market is crucial for economic self–sufficiency, health, feelings of belonging in and contributing to a new society (Asghari, 2025). Studies have shown both barriers and obstacles (e.g., Knappert et al., 2019; Moffitt et al., 2020; Senthanar et al., 2020) and facilitators (e.g., Barros de Alcantara Barros de Alcantara Hamrin, 2019; Dlamini et al., 2012; Koert et al., 2011) for immigrant women to enter the labor market in a new society.

Immigrants frequently face discrimination and racism in a new society, which negatively affects their perceived health (Lecerof et al., 2016; Manesis, 2014), particularly among immigrant women (Nakhaie & Wijesingha, 2014; Smedley, 2012). Health conditions are worsened by negative events, such as trauma, acculturation stress, and lack of social resources in the new surroundings, but vary greatly depending on sociocultural and economic circumstances, gender, generations, and the degree of social integration (Blomberg et al., 2024; Shawel Abebe et al., 2014).

For immigrant women, it takes a longer time to acquire a job in the new society compared to immigrant men (Backman et al., 2021; Kosyakova et al., 2023). Moreover, at work, these women risk facing double discrimination due to both ethnicity and gender (Aslan Akay & Ahmadi, 2022). Hence, studies elucidate that occupational health is challenging for immigrant employees (Mousaid et al., 2016; Sterud et al., 2018). Generally, immigrant employees face poorer working conditions, including psychosocial issues such as discrimination and bullying, as well as being more commonly exposed to different hazards in the physical working environment (Sterud et al., 2018; de Diego-Cordero et al., 2020). Especially vulnerable are immigrant women from lower–income countries, facing higher work–related health and safety risks than other groups of immigrants (de Diego-Cordero et al., 2020; Mousaid et al., 2016; Sterud et al., 2018; Saksena & McMorrow, 2020).

Indeed, learning the language and vocational training are facilitators for becoming part of a new society (Reinke & Goller, 2022), and volunteering serves as a context for informal learning and transition into the new society (Liu & Guo, 2021). Some immigrants participate, for instance, as mentees in mentorship programs and similar activities (Lai et al., 2017). Even though aiming to increase economic competitiveness, productivity, and employability, immigrants’ engagement in volunteering and requalification can be considered an effort to comply with the ruling relations (Liu & Guo, 2021). This, according to Liu and Guo (2021), deems immigrants’ knowledge and skills deficient and inferior, which risks further pushing immigrants into the dominant norms and values of the host society.

The assimilation discourse becomes even more exacerbated by several Western European countries assessing language and values tests as part of civic integration (Goodman & Wright, 2015). However, these requirements risk excluding some groups of immigrants. Jensen et al. (2021) displayed that differences in age, gender, and education significantly affect the capacity to become citizens. Women and older immigrants generally experience the civic integration process as more challenging (Jensen et al., 2021).

Workplace skills and knowledge of local language and culture are facilitators for integration (Knappert et al., 2019). Agency, such as seeking and seizing opportunities, attitudes, aspirations, flexibility, and adaptability, are significant personal aspects of the integration process (Verwiebe et al., 2019), aligning with the assimilation discourse. Immigrants are urged to be active, finding motivation and resources, and concurrently find strategies to cope with challenges (Knappert et al., 2019; Verwiebe et al., 2019).

### 1.1 A salutogenic perspective

There are several studies focusing on promoting health for immigrant women using a salutogenic perspective, but not directly addressing working life (cf. Bonmatí-Tomas et al., 2019; Hawkins et al., 2021). However, previous research on pathways to working life has primarily focused on barriers (Ertorer et al., 2022; Reinke & Goller, 2022). Studying health from a salutogenic perspective in relation to working life focuses on facilitators that enrich and promote employee health (Wiman et al., 2016) and promote the organizational environment (Mayer & Krause, 2013). A salutogenic perspective is a theory guiding health–promotion activities. From a salutogenic perspective, questions like promoting and maintaining good aspects are of interest rather than focusing on barriers and risks (Antonovsky, 1996). This perspective on health comprises mental, physical, and social health, and thus provides a holistic understanding of individuals’ state of health. The terms and concepts, such as a sense of coherence and generalized resistance resources, are useful in understanding a person’s mental health. This is especially true when a person has gone through tough difficulties and still seems to be healthy. Therefore, facilitating aspects for female immigrants’ establishment in working life are of interest in this literature review.

### 1.2 Rationale and aim

Altogether, although research shows that integration into working life in a new society is associated with complex processes wherein employment constitutes an important facilitator, there is a need for more studies that encompass aspects of pathways into working life for immigrant women. Since previous research has primarily focused on aspects connected to barriers and lack of resources for workplace integration (Ertorer et al., 2022), this study will focus on facilitators contributing to the integration into working life for women with immigrant backgrounds.

This literature review aimed to explore peer–reviewed research on facilitating aspects for immigrant women in their process of integrating into working life.

## 2 Materials and methods

### 2.1 The systematic approach

**Figure 1.**
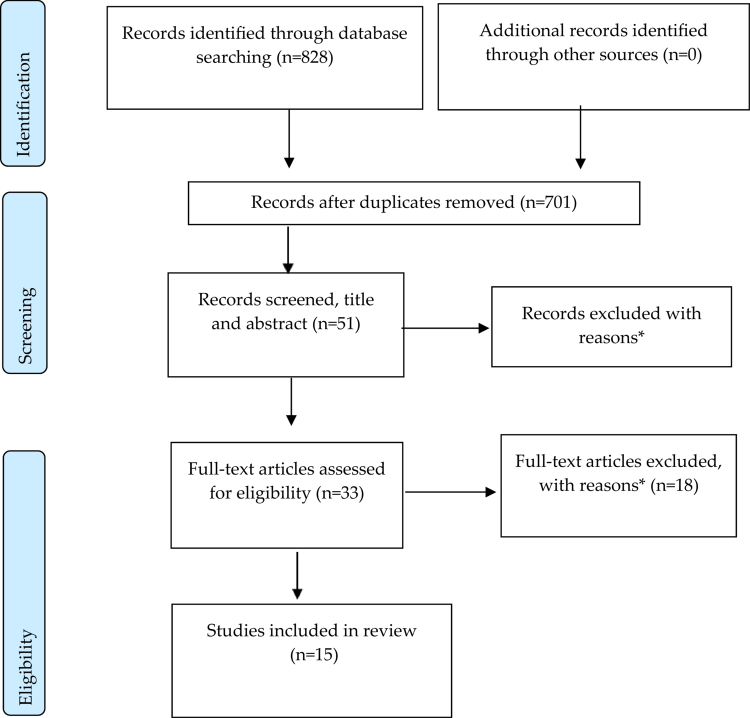
Prisma Flowchart of literature search and selection (Moher et al., 2009). * Reasons for exclusion: studies including both men–women and/or natives–immigrants, articles focusing on economic/socioeconomic aspects, policies, social and health care and services, mobility, wages, safety hazards, stressors, racism, precarity and exploitation of women, sex, drugs, children, HR aspects, management, abuse, second–generation immigrant, and segregation.

An integrative literature review was conducted to identify peer–reviewed research describing immigrant women and aspects facilitating their integration into working life. This broad review method includes searches for both experimental and non–experimental research and theoretical and empirical literature (Booth et al., 2016). The review was conducted according to the Preferred Reporting Items for Systematic Reviews and Meta–analyzes (PRISMA) (Moher et al., 2009). The PRISMA strategy includes the flow chart in Figure 1 and a checklist of items to include when reporting a systematic review.

Literature searches were conducted in the following databases: Web of Science Core Collection, Academic Search Elite, Scopus, ERIC, PsycINFO, PubMed, and Google Scholar during March 2024. A supplementary search in Web of Science Core Collection and Scopus was made in May 2025. Scopus is a multidisciplinary bibliographic database. ERIC is a database containing information in an educational context, and PsycINFO provides abstracts and citations to scholarly literature in the behavioral sciences and mental health literature. Literature search strategies were designed using thesaurus, MeSH (Medical Subject Headings), and free–text terms.

### 2.2 Criteria for inclusion

For inclusion, the research had to involve first–generation immigrant women, i.e., the results had to be explicitly linked to the women’s experiences, be published in English, and report on facilitating aspects of the integration process into working life. We excluded research on irrelevant populations, comparison studies, and articles published before 2010. No specific study design was excluded.

### 2.3 Search strategy

The screening was mainly conducted by the first author, and in a complementary screening, also the last author. A flowchart showing the outcome of the search and paper–selection process is provided in Figure 1. The search strategy is based on the building block strategy (SBU, 2017). As such, search terms and phrases have been used in blocks and searched separately. The blocks are combined in the final search. The blocks in this search strategy were workplace, women, immigrants, facilitators, and variations of these. See examples from two databases below.

Web of Science Core Collection was searched using the following keywords, limited to search in the abstract, summarized here as Boolean search mode: i) workplace* OR labor market AND ii) woman OR women OR female* AND iii) immigrant* OR migrant* OR refugee* OR newcomer* AND iv) facilitate* OR condition*. Scopus was searched using the following keywords, limited to search in title, abstract, and keywords, summarized here as Boolean search mode: i) workplace OR labor AND market AND ii) women OR female AND iii) immigrant OR refugee OR immigration AND iv) facilitators OR motivators OR enablers OR conditions.

The search encompassed several forms of immigration, including coming as a refugee or coming for another reason. In this paper, the definition of a migrant from the International Organization for Migration (IOM, 2024) is used: “a person who moves away from his or her place of usual residence, whether within a country or across an international border, temporarily or permanently, and for a variety of reasons”.

Searches of the online databases returned 828 records. After removing duplicates, the titles and abstracts of 51 records were screened. Most of these records came from Google Scholar (487), which did not match the inclusion criteria. Among the 51 screened articles (title and abstract), 18 articles were excluded. Inspection of the remaining 33 articles (full text) found that 15 articles met the criteria for inclusion. The articles were published between 2010 and 2024. Eleven of the articles were published in 2017 and later. The 15 articles are listed and summarized in Table I.

**Table I.** Characteristics of the articles included in the review.

Author country year of publication	Objectives/research questions	Method/setting participants	Article conclusion
Ng and Shan Canada 2010	How do individuals orient themselves to this discourse when they participate in the labor market?	Life–history–style interviews with 21 Chinese women.	To reiterate, critics of lifelong learning as state policy suggest that this discourse has become a new technology of governance serving to redefine the relationship between the state and the individual. The onus in neo–liberal globalization is to require individuals to take responsibility to keep up with economic and social change. The power of lifelong learning as an ideological and discursive frame is precisely its ability to render continual (re)education as an individualistic, natural and neutral phenomenon. This frame shifts attention away from the structure and nature of a post–Fordist and post–industrial economy (in the current era of globalization), which is characterized by a progressively flexible, non–permanent and disposable work force. By focusing on the experiences of workers themselves, in this case professional Chinese immigrant women in Canada, the results show how individuals take up this discursive frame in fitting themselves into a segmented labor market. The researchers further display how this frame intersects with other ideological and material practices to organize, reproduce and maintain gender and racial inequalities both in the labor market and in the society.
Koert, and Amundsen Canada 2011	What helps immigrant women workers to do well with changes affecting their work and what hinders them in that process? What would have been helpful to these workers to do well with these changes?	Interviews with 10 immigrant women.	A total of 182 incidents were extracted and grouped into 9 categories: personal beliefs/traits/values, taking action, skills/education, personal challenges, self–care, relationships/support, government/community resources, work environment, and contextual challenges. Results support and extend contentions that both internal/personal and external factors are key successful adaptation to change for immigrant women.
Dlamini, Anucha and Wolfe Canada 2012	Explore the sociopolitical benefits that immigrant women derive from their working experiences in their new country, the tensions and conflicts they may experience as they navigate workplace norms in their new country, and their strategies for reconciling these norms with those of their country of origin.	In–dept interviews with 37 women who had work experience in Canada.	Although some women described positive settlement experiences because of the support they received from friends and family members on their arrival (e.g., providing a temporary place to stay, introductions to agencies that provide English classes, assistance in finding a permanent home, and connections to their cultural community), the majority of women (26 of the 37) described struggles they had faced because of the lack of information or misinformation they had received before migrating about what Canada was like and what employment opportunities would be available.
Shan Canada 2015	Examination of how the neoliberal subjectivity of an entrepreneurial self is produced for some professional Chinese immigrant women in Canada. Explore how the women tried to reorient their career trajectory and optimize their employment opportunities after immigration.	Interviews 21 Chinese immigrant women in Canada.	As they strived for new career paths in Canada, the women demonstrated an incredible entrepreneurial spirit. They actively sought information, invested in training and education, and negotiated new identities and positionalities in the host labor market.To reach the social and professional status that they had in China, the women had to first of all make it through the highly competitive educational system, which has long been regarded as the route to social mobility for the masses. Since classical times, Confucianism has emphasized education as more valuable than anything else in the society.
Farcas and Gonçalves UK 2017	Explore the form of international mobility of Portuguese migrant workers in the United Kingdom. Explore which patterns of cross–cultural adaptation are more strongly associated with specific motives and forms of international mobility.	Interview via Skype with 50 Portuguese migrant workers (50% females).	Those who move abroad as self–initiated expatriates (SIEs) and assigned expatriates (AEs) explore career opportunities before leaving Portugal, while those who move abroad as immigrant workers (IWs) explore them after arriving in the United Kingdom. In addition, after moving abroad and beginning to explore the career opportunities, IWs who have a higher degree seemed to be more willing to accept jobs outside their qualification areas, contrary to SIEs, who explored career opportunities solely in their area of expertise, and before moving abroad. Therefore, the exploration of career opportunities varies by the form of international mobility.
Grigoleit–Richter Germany 2017	Although highly skilled migrants are privileged with regard to education, competencies, and abilities, the article argues that highly skilled migrant women’s transition into the labor market and their work performance are determined by the gendered and ethnicised conditions still prevalent in Science, Technology, Engineering, and Mathematics (STEM) fields.	Narrative–oriented interviews with 12 highly skilled migrant women, expert interviews with four recruitment agencies specialized in the placement of technical personnel, and expert interviews with various personnel from human resources or diversity programs of 10 corporations and 5 medium–sized enterprises in the field	Cultural prejudices and state regulations prevent companies from recruiting more international talent and moreover interfere with highly skilled migrants’ career mobility once they are gainfully employed in Germany. Nationality thereby determines which groups of migrants are allowed access to the labor market as well as the types of work and segments of the labor market they can engage in. Such immigration rules and regulations, policies, and interventions at the macro–level regulate and shape the experiences of highly skilled migrants. Contrary, to the discourse about the ‘best and brightest’ ethnicity and national origin has evidently not become any less important in the segment of highly skilled migrants.Despite their high level of education skills, and qualifications in engineering and technical training, which are the most transferable and, moreover, in high demand in Germany, their full participation and socio–economic mobility were delayed. The distinctive gender–segregation in the technology sector marked by differences in value systems and attitudes towards technology and technical skills caused a devaluation of their job performance. As this sector is oriented towards men and a masculine working habitus, migrant women were disadvantaged as their jobs and career paths are associated with specific gender stereotypes.
Villares–Varela Spain 2017	Analyze how the gendered and classed positions of middle–class and working–class migrant women business owners explain divergent meanings of becoming an entrepreneur and the absence of their spouses in running their businesses.	35 Latin American women entrepreneurs in Spain.	Middle–class Latin American immigrant women become entrepreneurs to promote their spouse’s career advancement while conforming to class–based norms of femininity. In contrast, lower class Latin American women view the business as a space of autonomy and occupational upward mobility that nevertheless also complies with working–class definitions of femininity.The examination of the trajectories of the Latin American immigrant women presented in this article challenge the claim that entrepreneurship per se will enable women entrepreneurs to engage in trajectories that provide self–realization and independence. The data indicate that women who independently operate their own business do not necessarily feel a sense of empowerment.
Cameron and Cabaniss USA 2018	Coupled with uncertain economic times, what motivates women, especially minority women, to invest in new businesses?	Fieldwork and semistructured interviews with enterpreneurs and community informants. 21 Hispanic women who owned a registered, for–profit enterprise.	When asked why they started their own businesses, participants offered three main explanations: (1) entrepreneurial succession, (2) employment opportunities and constraints, and (3) social ventures and passions. The research also highlights the role of agency in shaping entrepreneurial pursuits among a marginalized group. Despite the structural obstacles they faced as women and ethnic minorities, the Latinas in the study all made choices that enabled them to start their own businesses.
Chun and Cranford USA 2018	Examining the trajectory of Chinese immigrant women into homecare work in Oakland Chinatown.	In–depth interviews with 17 Chinese immigrant women.	Racism and language barriers in the general economy, coupled with family–based migration has created a densely–populated, regionally–specific community of Chinese immigrants in the Oakland Chinatown residential and commercial area, characterized by an aging immigrant population and ongoing contemporary migration flows. Many Chinese immigrant women found themselves working in a relatively new job sector in the co–ethnic economy, not because they chose the job but because the job “chose” them, as several interview participants pointed out explicitly.
Barros de Alcantara Hamrin Sweden 2019	Examine the experiences of immigrant employees with other organizational actors at two senior nursing units in Sweden.	Interviews with six female and three male immigrant nursing assistants living permanently in Sweden.	Trustful relationships with other organizational actors, during both formal and informal interactions, are considered essential facilitating inclusion of these immigrant workers. Immigrant workers experienced inclusion when they achieved language competence (or felt supported in their attempts to do so) and bridged cultural differences. The results also highlight conditions for interactions and leadership as factors influencing inclusion. In addition, inclusion implied acculturation or awareness of the values of native–born citizens.
Zani Taiwan 2020	Presenting the tactics used by Chinese women to overcome marginalization and exclusion in the labor market. This includes the exploration of novel markets, and the performance of digital labor and e–commerce, which produce new, networked, translocal, digitalized and emotional economies.	Ethnographic work and biographical interviews with 111 low–skilled Chinese migrant women.	Based on Wenfeng and her fellow migrants’ lived experiences, this article examined the creative digitalized economic practices produced and performed by Chinese migrant women at the crossroads among migrations, emotions, subalternity and virtual worlds. By drawing a cartography of women’s digitalized, networked and emotional economic practices, this analysis echoed Eva Illouz’s (2007) proposal, which conceives markets and economic action as being simultaneously socially and emotionally constructed. To answer the question put forward at the beginning of this article, the researcher found that novel imbrications among emotions, economies and digital platforms are being produced by migrants who can make good use of heterogeneous, translocal repertories of social and emotional resources to cope with the inequalities and hierarchies which characterize their migratory paths.Their online socializations, emotional exchanges and lowly–visible economic practices on WeChat illustrate the production of new forms of contestation of the social and economic inequalities which characterize the local and physical order. Therefore, when the latter becomes oppressive, positive and negative repertories of emotions support an inventive use of digital platforms, which can be transformed by actors into novel spaces for transgression, which sustain social mobility, economic independence and new aspirations.
Yeasmin, Koivurova and Kemppainen–Koivisto Finland 2022	Explore whether Social Entrepreneurship (SE) could be a measure that could facilitate the integration of immigrants, particularly immigrant women, into the Finnish labor market in the near future, with Lapland as a case study.	Focus group discussion, 50 persons in total.	According to the project, the co–operative is a low–risk and safe way for both immigrants and the long–term unemployed to learn about doing business. SE also provides access to leadership, management, finance and strategic development. The SE makes democratic decisions and at the same time develops its activities together. Immigrants integrate well through these activities, as the co–operative is a safety net for them, enabling them to combat stress and isolation from the labor market. Being part of a team also creates enthusiasm, creativity, a positive mood, and access to the job market though many different cultural clashes can also hinder teamwork to some extent, since they basically are from different cultural backgrounds. However, a positive team support in different mental turbulences enables them to overcome some of the challenges together.The SEs are in need of social knowledge, a resource–sharing forum, and a training event for its members for building their capacity for possible social networking through existing SEs in Lapland.
Zinatsa & Saurombe South Africa 2022	This research aimed at exploring the labor market experiences and self–initiated strategies of accompanying spouses, also referred to as tied migrants, in their attempt to achieve labor market integration (LMI) in South Africa.	The study used a qualitative research approach to interrogate the experiences of accompanying spouses in South Africa. Thirteen one–on–one interviews were conducted, each lasting for 1.5 h on average. Thematic analysis was applied to the data.	Self–initiated strategies that reflect agency and a pushback on governing technologies by accompanying spouses can facilitate integration into the South African labor market. However, these strategies do not guarantee full LMI. The broad exclusionary context, premised on ethnicised rationalities that characterize the South African labor market, makes full LMI difficult to achieve, particularly in the absence of support for integration.
Elitok & Nawyn USA ​​​2023	Existing research indicates that women’s careers are disadvantaged when they migrate for the purposes of a male partner’s career opportunities, but the mechanisms behind that pattern are not fully understood.	Interviews with 18 highly educated Turkish women who migrated to the US with their husbands to pursue a job or educational opportunity for their spouse	Over time, a process of re–domestication unfolded as the women’s priorities and identities as career–focused individuals shifted as a way to adapt to the limitations on their career advancement resulting from structural and cultural barriers to succeeding in the US labor market as dependent migrants. The women in the study understood that they were truncating their earlier professional goals, and they expressed dissatisfaction with that decision. But given the circumstances, they made the best choice they could with the options that they were given and chose to appreciate the benefits that they did experience.
Schieckoff Germany 2024	This study seeks to investigate the LFP (labor force participation) decision in its own right, exposing the gender–specific nature of this stage of labor market integration and its determinants, including labor market resources, like education and language skills, and motivations, like childcare pressures, gender role attitudes, and personality traits.	Data from the Recent Immigration Processes and Early Integration Trajectories in Germany (ENTRA) survey of recently–arrived immigrants.Respondents in the dataset range between the ages of 18 and 41. Excluding cases with missing information on the relevant variables, the analysis sample includes 1534 women (589 Poles, 415 Italians, 229 Syrians and 301 Turks).	The results highlight that, unlike immigrant men, for immigrant women, the LFP decision is much more salient, and both labor market resources as well as motivations are decisive determinants. Nonetheless, even after accounting for these factors, Turkish and Syrian women still show persistently lower LFP compared to immigrant women from European origins. Moreover, childcare responsibilities are identified as a crucial female–specific barrier.

### 2.4 Analytical procedure

Initially, the articles were read for an overall view of their content. Each article was then summarized in Table I including author, country, year of publication, objectives, methods, participants, and article conclusions. Characteristics of the studies included in the review are summarized in Table II. All authors extracted data from the sections of results, discussion, and conclusion to summarize and code the content. The PRISMA checklist (Moher et al., 2009) was used to extract data from articles. Extracted data were codified according to the qualitative content analysis method (Graneheim et al., 2017). The codes were initially compared to extract similarities and differences. Seven facilitators emerged in the selected articles. These aspects were initially analyzed into three tentative main categories. The authors then reviewed, discussed, and revised the tentative main categories into two main categories.

**Table II.** Characteristics of the studies included in the review.

		Number of studies
Year of publication	2010–2013	3
	2014–2017	4
	2018–2024	8
Country of settlement for participants	Canada	4
	Finland	1
	Germany	2
	South Africa	1
	Spain	1
	Sweden	1
	Taiwan	1
	UK	1
	USA	3
The continent of origin for participants^a^^,^^b^	Africa	3
	Asia	9
	Europe	7
	North America	1
	South America	3
	The Middle East	3
Design	Qualitative	14
	Quantitative	1
	Mixed method	0

^a^ The number of continents exceeds the number of studies included, as the sample may represent different continents in the same study.

^b^ Two studies did not state the continent of origin of the participants.

## 3 Results

Five facilitators emerged from the articles during the data analysis. These facilitators were analyzed into two main categories: the power of agency and social networks. The sub–categories in the power of agency category were active endeavors of learning culture and language, initiating inclusion and building personal capacity, and being entrepreneurial. In the social networks category, the sub–categories were supportive workplaces and supportive family members and friends. Table III shows the main categories and sub–categories found in the analysis of the included articles.

**Table III.** The main categories and the sub–categories from the analysis.

Main categories	Sub–categories
The power of agency	Active endeavors of learning culture and language
	Initiating inclusion and building personal capacity
	Being entrepreneurial
Social networks	Supportive workplaces
	Supportive family members and friends

### 3.1 The power of agency

One recurring pattern is the power of agency. The included studies indicate that women are proactive and innovative in their integration process and utilize a variety of options and strategies when searching for employment. However, structural factors could limit agency (Zinatsa & Saurombe, 2022). When experiencing low support for integration into working life, the women were forced to devise their own strategies. Strategies that are not always successful sometimes even were regarded as detrimental to long–term career trajectories (Zinatsa & Saurombe, 2022).

#### 3.1.1 Active endeavors of learning culture and language

Taking language classes and learning the language of the host society is one aspect of successful settlement (Barros de Alcantara Barros de Alcantara Hamrin, 2019; Dlamini et al., 2012; Farcas & Gonçalves, 2017; Grigoleit-Richter, 2017). One of the first steps to becoming part of the new society is to learn the language to make oneself understood and to understand simple everyday communication. The immigrant women invested time and effort in learning the language to get employment (Grigoleit-Richter, 2017). In order to communicate on the job, they had to learn the vocabulary, words, pronunciation, and expressions. Bilingual skills were also pinpointed as an advantage for immigrant women in the labor market (Cameron & Cabaniss, 2018). These skills are supportive in various situations, such as interpretation in business matters and in situations when newly arrived immigrants enter the workplace.

Some immigrant women had a higher education degree from their home country (Elitok & Nawyn, 2023; Grigoleit-Richter, 2017), some started their higher education in the host society, and others participated in programs offering educational and occupational training in the host country (Farcas & Gonçalves, 2017; Ng & Shan, 2010; Shan, 2015). Results showed the importance of having a university degree for both women from EU countries and non–EU countries (Schieckoff, 2024). Prior work experience did, however, not seem to play a significant role in women’s work–life participation. The women wanted to utilize their education and get qualified, skilled work. Many did not settle for simple, mundane employment but rather sought out new career possibilities and displayed an entrepreneurial mindset (Shan, 2015). However, some high–skilled women had to rethink and lower their career goals when coming to the new country (Elitok & Nawyn, 2023); however, they chose to appreciate the given options and choices.

The immigrant women encountered demands for increased awareness of local and cultural knowledge (Grigoleit-Richter, 2017). They developed different strategies to acquire skills and capabilities, such as different norms, habits, and social and cultural practices. This improved their chances to advance their careers and regain professional autonomy. Women from some countries were not used to employed work given that their home countries did not expect them to participate in the working life and contribute as citizens fully (Dlamini et al., 2012). These women strived to fit in, to learn how to become a citizen, and to receive employment in the host society despite their cultural backgrounds. Furthermore, Ng and Shan (2010) argued for the power of lifelong learning and its ability to render continual (re)education. A considerable proportion of the women in Ng and Shan (2010) study changed their careers because of the lengthy and costly recertification processes. These women wanted to align themselves with current labor market needs by investigating employers’ requirements. This knowledge could help them select an appropriate training program (Ng & Shan, 2010). One of the conclusions of Ng and Shan (2010) study was that the women’s active endeavors of learning indicated their resilience in a society that systematically devalued their social and economic worth.

Employers usually ask for work experience especially working in the host society. One strategy to achieve experience was to volunteer (Dlamini et al., 2012). In some cases, volunteer work led to paid employment, but others did not. Volunteering in a position related to their qualification area helped them find paid work. A physician in her home country volunteered in a nursing home and later got employment as a nursing aide (Dlamini et al., 2012). Due to the long time needed to get employment, the women were often forced to take any job offered to them even though the job was below their educational and/or professional qualifications (Dlamini et al., 2012).

#### 3.1.2 Initiating inclusion and building personal capacity

Moving to another country requires agency (Farcas & Gonçalves, 2017) to live in the new society and take part in agency programs for immigrants, e.g., learning to write a CV (Dlamini et al., 2012). The women found it helpful to remain active, e.g., by retraining and networking and taking steps towards workplace integration (Koert et al., 2011). Here, the most significant aspect was that the women themselves were active and sought out the necessary skills and resources. Barros de Alcantara Hamrin (2019) highlighted that inclusion depends significantly on the immigrants’ willingness to be integrated, to take initiative, and to do their job. Working on bridging cultural differences in their daily interactions with colleagues could impact integration.

Women actively sought information (Shan, 2015), invested in education, and negotiated new identities and opportunities to get a job. In some cases, immigrant women had to rethink and change some of their beliefs and practices to fit in at the workplace (Dlamini et al., 2012). For example, several had neither worked with men nor worked night shifts. They had to reframe their work experience (Dlamini et al., 2012). When values and attitudes differed and disagreed, immigrant women preferred to maintain values they learned in their home country. Norms that they had grown up with including respect for the elderly, not breaking agreed rules, and not gossiping, were considered important to maintain in meeting other people (Barros de Alcantara Hamrin, 2019). The women did not want to imitate the negative behavior they found among citizens in the host society.

Inner strength, resilience, and flexibility are examples of internal resources for doing well with changes that immigrant women could meet (Koert et al., 2011). They also talked about spiritual faith and feelings of hope as helping aspects; self–care is necessary for body, mind, spirit, and emotions (Koert et al., 2011). The women were active in building their capacity and could cope with changes that affected their work and their need to work. This resulted in the women feeling in control and growing in self–esteem and self–confidence (Koert et al., 2011).

#### 3.1.3 Being entrepreneurial

Social entrepreneurship can serve as a safe business network where women can develop their skills through community involvement (Yeasmin et al., 2022). The path to entrepreneurship was driven by a personal passion and/or commitment to a social problem (Cameron & Cabaniss, 2018). Entrepreneurs desire personal fulfillment and/or social change, such as some women’s passion for food and culture, which made them start their businesses (Cameron & Cabaniss, 2018). Photography, assisting others with computers, interpretation, and design services are other examples of businesses that evolved through entrepreneurship. One woman found a unique niche and started a business for Spanish speakers who had difficulties obtaining design services due to language barriers (Cameron & Cabaniss, 2018). She could assist them due to her language skills and her cultural knowledge.

Class and ethnicity were crucial in understanding immigrant female entrepreneurs (Villares-Varela, 2017). Villares-Varela (2017) concluded that sensitivity to class (re)positioning and context seem to be crucial in understanding the trajectories and experiences of migrated women entrepreneurs. Hence, to provide immigrant women with the required support, the organizational actors must be regarded as trustworthy and reliable and need to possess the requisite knowledge.

### 3.2 Social network

Successful integration at the workplace depends on leadership and management. Trust and good relationships were key aspects. The support from family and friends was helpful for the women, especially during the first time after arrival. The social network was extended after some duration in the host society.

#### 3.2.1 Supportive workplaces

A supportive leader at the workplace was often considered significant, and the inclusion was based on cooperation among colleagues regardless of their origin (Barros de Alcantara Hamrin, 2019). Other external aspects were organizational conditions, such as creating space and time to interact, along with adequate leadership for ethnically diverse groups. The leaders at the workplace encouraged the immigrant women to ask for help. When the co–workers trusted each other, they could ask for and receive information from more experienced co–workers–preferably other immigrants.

One woman described how construction industry contacts “helped me feel like a part of an industry here and like I was taking a positive step, like kind of when you are being proactive, you feel good, and then it helps you feel more confident at work” (Koert et al., 2011*,* *p*. 200).

Supervisors and leaders became more supportive when they saw that the women were working hard and could be trusted (Dlamini et al., 2012). The women felt acknowledged at the workplace and that the leaders trusted them to do their work well. Supportive supervisors and leaders led to better relationships, and thus, the work environment improved (Dlamini et al., 2012).

The women found that trustful relationships with organizational actors, in formal and informal interactions, were helpful in the integration process (Barros de Alcantara Hamrin, 2019). There may be several actors and organizations the women encounter in the host society on their journey to employment, e.g., employers in workplaces and people in governmental and non–governmental organizations.

#### 3.2.2 Supportive family members and friends

Family and friends' support on time of the women’s arrival was positive for the settlement experiences (Dlamini et al., 2012). This support could include providing a temporary place to stay, introductions to agencies providing language classes, assistance in finding a permanent home, and connections to their cultural community. Most women found employment through recommendations from friends (Dlamini et al., 2012).

The women initiated different informal social networks (Chun & Cranford, 2018), which became channels for information between newcomers and immigrants who had lived longer in the host society. Immigrant women gathered and created “emotional communities” in novel digital spaces (Zani, 2020). Here, they shared experiences, advice, suggestions, and emotions. This also led to offline activities. WeChat groups (Zani, 2020) were a place for women to share their feelings about their jobs. The shared emotions resulted in interactions and affections that could challenge and transform the rigidity of women’s social positions (Zani, 2020). The social network supported the women in coping with inequalities and hierarchies, which characterized their paths of migration (Zani, 2020). Women also found their business inside this online group with several clients. The women called each other “sisters.” They knew each other’s social, cultural, and emotional preferences. As a result, one woman started a virtual market for traditional Sichuanese food (Zani, 2020). She used her feelings for her “sisters,” her knowledge, and her skills in cooking to do business. By doing this, the feelings of belonging and connectedness increased.

## 4 Discussion

This paper systematically reviewed the facilitating aspects of immigrant women's pathways into working life. The results show two main categories: the power of agency and social networks. The power of agency and initiatives to be integrated was prominent in the results (Barros de Alcantara Hamrin, 2019; Farcas & Gonçalves, 2017; Koert et al., 2011). The women actively searched for the required information. They invested time and effort in learning and training. They negotiated new identities to become citizens in the new country. Similarly, rights and citizenship are the foundation of Ager and Strang (2008) conceptual framework, defining core domains of integration. Learning the language was a significant investment and an essential aspect of a successful settlement, which is in line with former studies (Barros de Alcantara Hamrin, 2019; Dlamini et al., 2012; Farcas & Gonçalves, 2017; Grigoleit-Richter, 2017). Education and active endeavors of learning, e.g., work skills, norms, and social and cultural practices, characterized women who successfully found their places in the new society (Barros de Alcantara Barros de Alcantara Hamrin, 2019; Dlamini et al., 2012; Farcas & Gonçalves, 2017; Grigoleit-Richter, 2017; Ng & Shan, 2010; Shan, 2015). A lack of knowledge of the host country’s language is a source of misunderstanding (Knappert et al., 2019). In line with the results, Ager and Strang (2008) discuss language and cultural knowledge as facilitators for integration.

The results further showed that adequate language skills often are required in the host society, e.g., for getting a job (Barros de Alcantara Hamrin, 2019; Dlamini et al., 2012; Farcas & Gonçalves, 2017; Grigoleit-Richter, 2017). Several European countries require sufficient knowledge in language tests to receive permanent residence permits and improve the conditions for long–term successful integration into the labor market (Vink et al., 2021). However, delayed integration was often observed for migrants with lower levels of education after introducing language requirements (Vink et al., 2021). Class differences, as well as age and gender differences, affect immigrants’ capacity to become citizens (Jensen et al., 2021). As elucidated in previous research, women from lower–income countries are facing higher work–related risks compared to other groups of immigrants (de Diego-Cordero et al., 2020; Mousaid et al., 2016; Sterud et al., 2018; Saksena & McMorrow, 2020). Goodman and Wright (2015) questioned whose interests–the immigrant or the state–are being served by this new paternalism. In all cases, Jensen et al. (2021) emphasized that integration “… is not a matter of immigrants’ desire, willingness or honest effort to contribute and become competent citizens. It depends on qualifications and human resources, which some people simply are incapable of acquiring” (*p*. 1062). When citizenship is delayed, the entire integration process is put on hold since the foundation of integration is rights and citizenship (Ager & Strang, 2008).

Participating in language classes and learning the language of the host society was considered meaningful and important (Barros de Alcantara Hamrin, 2019; Dlamini et al., 2012; Farcas & Gonçalves, 2017; Grigoleit-Richter, 2017). This aligns with the findings of Esses et al. (2017), Lai et al. (2017) and Moffitt et al. (2020), which indicate that immigrants must prioritize learning the local language and securing employment or other support possibilities with the same urgency as finding accommodation. Employment is indeed connected to health, independence, and a sense of coherence in a new society (Ager & Strang, 2008; Antonovsky, 1996; Moffitt et al., 2020). Immigrant women encountered demands for increased awareness of local and cultural knowledge and fluency in the local language (Grigoleit-Richter, 2017). Reducing barriers to cultural proximity as a positive consequence of education is key to promoting successful integration (Backman et al., 2021). The learning requests highlighted in the data align with those found in other studies (e.g., Knappert et al., 2019; Moffitt et al., 2020). More specifically, the women were active in their learning process, not only to learn the language and the necessary skills for the workplace but also to learn the new culture. In supporting these processes, studies show that a salutogenic perspective is helpful to promote social inclusion in general (Bonmatí-Tomas et al., 2019; Hawkins et al., 2021). Given the health challenges (Mousaid et al., 2016; Sterud et al., 2018) and risks of discrimination (Nakhaie & Wijesingha, 2014; Smedley, 2012) immigrant women face in working life, a salutogenic focus on facilitators that enrich and promote employee health (Wiman et al., 2016) and promote the organizational environment (Mayer & Krause, 2013) is crucial in connection to the workplace integration.

Several professional associations have implemented requirements for the recertification of foreign–trained persons to exert control over the professions, according to the results of Ng and Shan (2010). However, due to the complexity of the recertification process, female immigrants chose another pathway instead. Changing their employment trajectories was partially accomplished through a market rationale underpinning the lifelong learning discourse (Ng & Shan, 2010). In other research, Liu and Guo (2021) indicated that this view of lifelong learning–including deskilling and requalification–further pushes the immigrants into the dominant norms and values of the host society. In these results, however, the women found it advantageous to incorporate the idea of lifelong learning (Ng & Shan, 2010) and see their human capital as a facilitator and advantage, as Knappert et al. (2019) emphasized in previous research.

The results further indicated that one important aspect of advancing careers and regaining professional autonomy was developing different strategies to acquire skills and capabilities, such as in norms, habits, and social and cultural practices (Grigoleit-Richter, 2017). Another strategy shown in the results was that volunteering in a position related to their qualification area helped women find paid work (Dlamini et al., 2012). Other studies have also shown volunteering to be an important source of immigrants’ transition into the new society (Liu & Guo, 2021; Pelters et al., 2021). Collectively, volunteering and lifelong learning concerning recertification can reinforce assimilation from a neoliberal economic perspective–this contrasts with the desired inclusion in the host society.

The process of integration, with cultural and language learning, demands women’s agency and negotiating parts of the culture of origin (Dlamini et al., 2012). On the other hand, the absence of support for integration could lead to self–initiated strategies, not always beneficial to long–term career trajectories (Zinatsa & Saurombe, 2022). Women felt that misunderstandings in the workplace often happened due to differences in culture (Barros de Alcantara Hamrin, 2019), caused by differences in the prevailing workplace culture. However, previous research has shown that the natives tended neither to engage in integration processes nor to take sufficient responsibility for the successful integration of their immigrant co–workers (Malik & Manroop, 2017). As a result, some women had to rethink and change some of their beliefs and practices to fit in at the workplace. It was crucial to obtain a job and fit in with the perceived values of the host society (Dlamini et al., 2012). This quest to fit in, Dlamini et al. (2012) continue, requires learning about the work culture in the host society, the expectations of women in the workforce, and reframing previous work experience. An earlier study also emphasized this issue (Lai et al., 2017). Hence, there are barriers in the workplace due to diverse cultures.

Lai et al. (2017) described immigrants’ lack of knowledge and understanding of the culture in the host society and of values and unwritten rules. Some women did not want to embrace all the new values because they considered the previous values learned in their home country to be more human (Barros de Alcantara Hamrin, 2019). Previous research (Moulettes, 2021) concluded that integration is doomed to fail if people of different nationalities, ethnicities, ideologies, and religions need to tone down important aspects of their identity and consequently adapt and assimilate to dominating discourses. According to Penninx and Garcés-Mascareñas (2016), important aspects such as reciprocal learning in a mutual integration process would risk being lost. Policies in many countries emphasize a two–way directed integration process (Malik & Manroop, 2017). There can be practical tensions between a host society’s expectations of immigrants assimilating into the working life and the immigrants’ possibilities of contributing with all their backgrounds, experiences, and competencies.

Immigrant women with previous experience working in a multicultural environment could better identify with the new country’s culture (Farcas & Gonçalves, 2017). However, the results from Schieckoff (2024) showed less importance of prior work experience. On the contrary, holding a university degree facilitated integration into working life. The women negotiated a form of hybrid identity (Barros de Alcantara Hamrin, 2019) and assimilated characteristics of the host society, supporting them to regain some of their lost human capital, control, and power. This is consistent with previous research, which means that human capital (qualifications and skills) can become a facilitator at the individual level (Knappert et al., 2019). In the results, inner strength, resilience, and flexibility are examples of internal resources for coping with changes (Koert et al., 2011). The women also mentioned spiritual faith, hope, and the importance of caring for themselves in terms of body, mind, spirit, and emotions (Koert et al., 2011). These positive aspects, internal resources, were the women’s generalized resistance resources and consequently enhanced a sense of coherence (Antonovsky, 1996, Antonovsky, 2005). The results indicated that family and friends were important networks onarrival to the new country (Dlamini et al., 2012), to get a place to call home, and later make contacts in the labor market. In previous research, Ager and Strang (2008) emphasized housing, health, education, and employment as aspects supporting the integration process. Trustful relationships with leaders and co–workers lead to a satisfactory working life and are important for integration at an organizational level, as shown by Barros de Alcantara Barros de Alcantara Hamrin (2019).

The importance of the immigrants’ agency, adaptation, and activity in their integration process is a prominent pattern in the results (Dlamini et al., 2012; Farcas & Gonçalves, 2017; Koert et al., 2011). Some immigrant women began a new career in social entrepreneurship, developing their skills through community involvement (Yeasmin et al., 2022). The immigrants’ willingness to be integrated and take initiative also influenced the process of integration (Barros de Alcantara Hamrin, 2019). Participation in civil society activities could be an important factor in promoting health and integrating into society and the working life (Kostenius et al., 2021; Pelters et al., 2021). The OECD (2016) argues that integration policies will likely be ineffective without civil society organizations, a welcoming business environment, and the support of local communities. Emphasizing integration as a two–way process involves the host society creating opportunities for the immigrant’s full economic, social, cultural, and political participation and adaptation and activity by migrants (European Commission, 2020).

## 5 Conclusions and considerations for the future

The key source of successful integration discovered in this study is the inherent power of agency and taking necessary initiatives, which include actively learning the culture and language, starting inclusion, and developing personal capacity. Another prominent result is the expectations from the host society of the immigrants’ assimilation, rather than workplaces and natives contributing to the inclusion. To conclude, there seems to be an imbalance between the discourse of integration as a two–way process and the idea of assimilating to dominant ideas of workplaces and society. This echoes findings from Ertorer et al. (2022) in a Canadian context. Consequently, there is a risk of losing valuable working experiences and skills possessed by immigrant women. This imbalance needs to be addressed at a governmental policy level, by employers and other societal stakeholders. Based on a salutogenic approach, there is a need for further interventions focusing on facilitators to support female immigrants on their pathway to working life in a new society (cf. Bonmatí-Tomas et al., 2019). The review has provided insight into research in the field of immigrant women and facilitators on their way to finding work and employment in the host society. Even though facilitators were focused on the current study, every facilitator has a negative side or a “lack of…”. Further research is needed, addressing both aspects and resources facilitating and impeding workplace integration for immigrant women.

## 6 Study limitations

One limitation of this study was that only 15 articles matched the inclusion criteria. The literature search, however, was conducted in several databases and with broad search terms. However, additional keywords could have been included as search terms for facilitators and enablers, such as *circumstances* and *encouragers*. One feasible way to overcome this limitation would have been to perform a scoping review (Booth et al., 2016), which allows the inclusion of unreviewed research (gray literature) such as ongoing research, conference abstracts, governmental or quasi–governmental reports, reports from key organizations, and information from key websites in the field of interest. Another limitation might be the focus on facilitators. This focus excludes aspects that impede workplace integration. Yet a limitation may be the small distribution of geographical diversity. All studies, except two, were conducted in the Western world. However, these geographical areas receive many immigrants. Nevertheless, the results in the included studies do not differ significantly despite the relatively limited geographical differences. Altogether, this integrative literature review of female immigrants' establishment in the labor market demonstrates that the included studies were derived from different countries. It is reasonable to suggest that the contexts and cultures these female immigrants are coming from and the host country ´ s cultural contexts play a significant role in the establishment strategies the females embrace. Despite this, we found aspects in the studies considered common tendencies. Hence, the findings in the studies within the sample show common patterns in female immigrants ´ subjective experiences, forming the point of departure for the results in this paper.

## Supplementary Material

Supplementary Material PRISMA 2020 Checklist

## Data Availability

Data sharing is not applicable to this article as no new data were created or analyzed in this study.
